# English Opinion of American Medical Association

**Published:** 1874-11

**Authors:** 


English Opinion of the American Medical Association.—The editor of the London *Medical Press*, in giving an outline of the proceedings of the Medical Association, which was in session at the same time as the British, remarks : "We do not propose to contrast the two associations, as we should find our own to be rather inferior in many respects." This sentiment is noteworthy in view of the depreciatory strictures of a few of our own journalists, one of whom actually hoped that physicians abroad would not regard the members of the association as a fair representation of the profession in America.

